# *Klebsiella pneumoniae* infection causes mitochondrial damage and dysfunction in bovine mammary epithelial cells

**DOI:** 10.1186/s13567-021-00898-x

**Published:** 2021-02-10

**Authors:** Jia Cheng, Jv Zhang, Jingyue Yang, Bing Yi, Gang Liu, Man Zhou, John P. Kastelic, Bo Han, Jian Gao

**Affiliations:** 1 Department of Clinical Veterinary Medicine, College of Veterinary Medicine, China Agricultural University, Beijing, 100193 China; 2 Department of Production Animal Health, Faculty of Veterinary Medicine, University of Calgary, Calgary, AB T2N 4N1 Canada

**Keywords:** *Klebsiella pneumoniae*, Mitochondrial damage, bMECs, ROS, Calcium

## Abstract

*Klebsiella* *pneumoniae*, an important cause of bovine mastitis worldwide, is strongly pathogenic to bovine mammary epithelial cells (bMECs). Our objective was to determine the role of mitochondrial damage in the pathogenicity of *K. pneumoniae* on bMECs, by assessing several classical indicators of mitochondrial dysfunction, as well as differentially expressed genes (DEGs). Two *K. pneumoniae* strains (HLJ-D2 and HB-AF5), isolated from cows with clinical mastitis (CM), were used to infect bMECs (MAC-T line) cultured in vitro. In whole-transcriptome analysis of bMECs at 6 h post-infection (hpi), there were 3453 up-regulated and 3470 down-regulated genes for HLJ-D2, whereas for HB-AF5, there were 2891 up-regulated and 3278 down-regulated genes (*P* < 0.05). Based on GO term enrichment of differentially expressed genes (DEGs), relative to the controls, the primary categories altered in *K. pneumoniae*-infected bMECs included cellular macromolecule metabolism, metabolic process, binding, molecular function, etc. Infections increased (*P* < 0.05) malondialdehyde concentrations and formation of reactive oxygen species in bMECs. Additionally, both bacterial strains decreased (*P* < 0.05) total antioxidant capacity in bMECs at 6 and 12 hpi. Furthermore, infections decreased (*P* < 0.05) mitochondrial membrane potential and increased (*P* < 0.01) mitochondrial calcium concentrations. Finally, severe mitochondrial swelling and vacuolation, as well as mitochondrial rupture and cristae degeneration, were detected in infected bMECs. In conclusion, *K. pneumoniae* infections induced profound mitochondrial damage and dysfunction in bMECs; we inferred that this caused cellular damage and contributes to the pathogenesis of *K. pneumoniae*-induced CM in dairy cows.

## Introduction

Bovine mastitis caused by pathogenic microorganisms is an important economic threat to the dairy industry [1]. *Klebsiella pneumoniae,* a major pathogen causing clinical mastitis (CM), induces a strong immune response, altering milk quality and causing inflammatory changes in the udder [2–4]. Furthermore, CM caused by *K. pneumoniae* is usually more severe than CM due to *E. coli* [3]. Substantial damage of mammary tissue, specifically epithelial cells, occurs in CM caused by *Klebsiella* [5, 6].

Mitochondria are intracellular organelles with multiple features due to their bacterial ancestry [7]. In addition to being a cell’s energy powerhouse, they are also a reservoir for cellular Ca^2+^, which is crucial for cell life and death [8]. Furthermore, mitochondria have a role in the innate immune system by harboring innate sensors that can detect bacteria and trigger immune activation, making them an attractive target for pathogens [9, 10]. Indeed, during co-evolution of pathogens and hosts, the former have developed strategies to manipulate cellular machinery, particularly subverting mitochondrial function [11] and commonly causing damage to these organelles. Mitochondrial damage is usually characterized by increases in calcium and reactive oxygen species (ROS), a decrease in mitochondrial membrane potential (MMP), and morphological damage [12, 13]. In addition, a lower MMP and decreased activity of the respiratory chain with a simultaneous increase in ROS production, can lead to mitochondrial dysfunction [14].

In our previous study, *K. pneumoniae* isolated from bovine mastitis caused apoptosis and pro-inflammatory responses of bMECs cultured in vitro [15]. *K. pneumoniae* isolates adhered to and invaded bMECs, causing ultrastructural damage, including swollen mitochondria and vesicle formation on the cell surface. In addition, in infected bMECs, there were increases in transcriptional expression of interleukin-1β (IL-1β), interleukin-6 (IL-6), interleukin-8 (IL-8) and tumor necrosis factor-α (TNF-α) genes and production of IL-1β, IL-8 and TNF-α [15]. However, whether mitochondrial damage has a critical role in pathogenicity of *K. pneumoniae* on bMECs has not been well established. Therefore, our objective was to determine the role of mitochondrial damage in the pathogenicity of *K. pneumoniae* on bMECs by assessing several classical indicators of mitochondrial dysfunction, as well as differentially expressed genes (DEGs).

## Materials and methods

### *Klebsiella pneumoniae* strains

Two *K. pneumoniae* strains (HB-AF5 and HLJ-D2) isolated from cows with CM were used. Both of these strains belong to the K57 capsule serotype and have *entB*, *ybtS*, *iutA*, and *mrkD* genes. However, HLJ-D2 is a hypermucoviscous (HMV) strain, whereas HB-AF5 is a non-HMV strain [15, 16]. In our previous study, these two *K. pneumoniae* strains were cytopathogenic on bMECs and induced pro-inflammatory responses [15].

### Cultured bovine mammary epithelial cells (bMECs)

A line of bMECs (MAC-T; Shanghai Jingma Biological Technology Co., Ltd., Shanghai, China) was cultured in cell culture plates (Corning Inc., Corning, NY, USA) in Dulbecco’s modification of Eagle’s medium (DMEM) with high glucose (Hyclone Laboratories, Logan City, UT, USA), supplemented with 10% heat-inactivated fetal bovine serum (FBS; Gibco, Grand Island, NY, USA) at 37 °C with 5% CO_2_. In all experiments, cells were allowed to grow and adhere for 24 h in culture medium prior to being infected with *K. pneumoniae*. At 70 to 80% confluence, bMECs were seeded in 6- or 96-well plates and incubated in 5% CO_2_ at 37 °C. Once bMECs reached 80 to 90% confluence, they were challenged with *K. pneumonia*e (three wells with bMECs + HB-AF5 and three wells with bMECs + HLJ-D2), whereas control cells were cultured only with culture medium (three wells with only bMECs).

### Cell infections

Before infection, bMECs in a well were stained with 0.4% trypan blue (Beyotime Biotechnology, Beijing, China) and enumerated with a cell counter. The *K. pneumoniae* cultures were centrifuged for 15 min at 6000 *g*, washed once in sterile Phosphate Buffered Saline (PBS, Beijing Solarbio Science & Technology Co., Ltd., Beijing, China) at pH 7.2, and re-suspended in DMEM, with concentrations adjusted to achieve multiplicity of infection (MOI) = 5. The MOI was defined as the ratio of added *K. pneumoniae* to bMECs.

### RNA isolation

At 6 hpi, bMECs (plus non-infected controls) were collected and used for RNA sequencing analyses. Briefly, bMECs were washed with PBS and harvested with 1 mL of Trizol to extract total RNA, according to the manufacturer’s protocol (TransGen Biotech Co., Ltd, Beijing, China). Total RNA yield and purity were determined by absorbance at 260 and 280 nm, using a NanoDrop-2000 spectrophotometer (Thermo Fisher Scientific Inc., Waltham, MA, USA).

### RNA sequencing

For RNA sequencing, 2 µg of RNA per sample was used. Sequencing libraries were generated using NEBNext UltraTM RNA Library Prep Kit for Illumina (NEB, Ipswich, MA, USA) following manufacturer’s recommendations, with index codes added to attribute sequences to each sample. Clustering of the index-coded samples was performed on a cBot Cluster Generation System using TruSeq PE Cluster Kit v4-cBot-HS (Illumina, San Diego, CA, USA), according to the manufacturer’s instructions. After cluster generation, the library preparations were sequenced on an Illumina Hiseq 4000 platform and paired-end 150 bp reads were generated. Differential expression analysis of the two infected groups (HB-AF5 and HLJ-D2) and the control group was performed using the DESeq R package (Version 1.10.1), which provides statistical routines for determining differential expression in digital gene expression data, using a model based on the negative binomial distribution. The resulting *P* values were adjusted using Benjamini and Hochberg’s approach [17] for controlling the false discovery rate. Genes with an adjusted *P*-value < 0.05, as detected by DESeq, were designated as being differentially expressed. Gene Ontology (GO) enrichment analysis of differentially expressed genes (DEGs) was implemented by the GOseq R package-based Wallenius non-central hyper-geometric distribution [18] that adjusts for gene length bias in DEGs. MapMan software was used to analyze differential expression in metabolic pathways [19]. Heat maps were generated using the gplots package in R [20]. To identify genes that were significantly up- or down-regulated in infected bMECs compared to the corresponding non-infected control, DeSeq2 was used to generate log_2_ fold change values for each gene. Volcano plots were used to provide a global view of distribution of DEGs in each group. In addition, to characterize the functions and pathways of genes obtained from RNA-Seq, functional annotation and classification were performed by comparing sequences with Gene Ontology (GO) databases [21].

### Malondialdehyde (MDA) and total antioxidant capacity (T-AOC)

To determine effects of *K. pneumoniae* on the antioxidant status of bMECs, malondialdehyde (MDA) concentrations and total antioxidant capacity (T-AOC) in supernatant fluid of cultured bMECs were determined using commercial kits, in accordance with the manufacturer’s instructions (Nanjing Jiancheng Technology Co. Ltd., Nanjing, China). The bMECs were cultured in six-well plates and challenged with HB-AF5 or HLJ-D2 *K. pneumoniae* (MOI = 5)*,* with sampling after 1, 3, 6 and 12 h. Noninfected cells were similarly incubated as a control and sampled concurrent with infected bMECs.

Concentrations of MDA were determined using the thiobarbituric acid (TBA) method. The MDA detecting liquid (containing TBA) was prepared according to the manufacturer’s instructions. After infection with HB-AF5 and HLJ-D2 *K. pneumoniae*, 200 µL of bMECs supernatant was collected from each well, put into centrifuge tubes and centrifuged (8000 × *g*, 5 min). After centrifugation, 100 µL of supernatant was collected, 200 µL MDA detecting liquid added and the resulting solution thoroughly mixed. The tube was placed in boiling water for 15 min, then cooled to room temperature and centrifuged (1000 × *g*, 10 min). The supernatant was assessed at 532 nm (680 Multipurpose Microplate Reader, Biorad, Hercules, CA, USA). Assay results were normalized to the protein concentration in each sample and expressed as nmol/mg protein.

The T-AOC was determined using the 2, 2′-azino-bis (3-ethylbenzthiazoline-6-sulfonic acid) (ABTS) method, using commercial kits. The ABTS detecting liquid and a standard curve were prepared according to the manufacturer’s instructions. At 1, 3, 6 and 12 hpi with HB-AF5 or HLJ-D2 *K. pneumoniae* (MOI = 5), bMECs were scraped off, washed with PBS three times, put into 200 µL cold PBS and ultrasound homogenization was done to disrupt cells and release antioxidants. After centrifugation (12 000 × *g*, 5 min, ~4 °C), 10 µL of supernatant was put into the wells of a 96-well plate, 200 µL ABTS detecting liquid added and after 5 min, absorbance was measured at 405 nm (680 Multipurpose Microplate Reader, Biorad).

### Reactive oxygen species (ROS)

Generation of ROS was monitored with a Reactive Oxygen Species Assay Kit (Beyotime Institute of Biotechnology, Shanghai, China) using a 2′, 7′-Dichlorodihydrofluorescein diacetate (DCFH-DA) fluorescent probe. DCFH-DA changes into fluorescent dichlorofluorescein (DCF) when intracellular DCFH reacts with ROS. Cultured bMECs in six-well plates were challenged with HB-AF5 or HLJ-D2 *K. pneumoniae* (MOI = 5). Noninfected cells were similarly incubated as a control. At 1, 3, 6 and 12 hpi, bMECs were washed with PBS and stained with DCFH-DA. According to the instructions, the DCFH-DA was diluted to 10 μM in fresh DMEM medium and incubated with bMECs for 30 min at 37 °C in the dark. As a positive control, untreated bMECs were incubated with rosup (50 µg/µL) for 30 min and then stained with DCFH-DA, whereas for a negative control, untreated bMECs were stained with DCFH-DA. Within 1 h, DCFH-DA fluorescence was detected with an inverted fluorescence microscope at 488 nm excitation and 525 nm emission using a laser scanning confocal microscope (Olympus FV1200, Olympus Corporation, Tokyo, Japan).

### Mitochondrial membrane potential (MMP)

A fluorescent carbocyanine dye JC-I assay kit (Beyotime Institute of Biotechnology, Shanghai, China) was used to determine MMP. Cultured bMECs in six-well plates were challenged with HB-AF5 or HLJ-D2 *K. pneumoniae* (MOI = 5), with noninfected cells similarly incubated as a control. At 1, 3, 6 and 12 hpi, bMECs were washed and re-suspended in PBS. Then, 0.5 mL of JC-I PBS buffer solution (10 µg/mL) was added to the suspension, which was kept in darkness at 37 °C for 30 min. After washing to remove excess probe, MMP was measured using flow cytometry (Becton Dickinson, Bergen City, NJ, USA) and presented as a relative ratio.

### Cytoplasmic calcium concentration

Cytoplasmic calcium concentrations in bMECs were determined with a fluo-3 assay kit (Beyotime Institute of Biotechnology, Shanghai, China). Cultured bMECs in six-well plates were challenged with HB-AF5 or HLJ-D2 *K. pneumoniae* (MOI = 5), with noninfected cells similarly incubated as a control. At 1, 3, 6 and 12 hpi, bMECs were washed three times in PBS, detached with Trypsin–EDTA solution and re-suspended in DMEM (with 10% FBS). Then, bMECs were centrifuged at room temperature for 10 min at 1000 × *g*, washed with Ca^2+^-free Krebs Solution (with 4 mM EGTA in lieu of CaCl_2_) and stained with fluo-3 (final concentration, 10 μM) and incubated at 37 °C for 30 min in the dark. Fluorescence of the cell suspensions was analyzed with flow cytometry (BD FacsCalibur flow cytometer, Becton Dickinson) and Ca^2+^ concentration expressed as mean fluorescence of fluo-3.

### Mitochondrial calcium concentration

Cultured bMECs in six-well plates were challenged with HB-AF5 or HLJ-D2 *K. pneumoniae* (MOI = 5), with noninfected cells similarly incubated as a control. At 1, 3, 6 and 12 hpi, bMECs were washed with PBS, detached with trypsin–EDTA solution and centrifuged at room temperature for 10 min at 1000 × *g*. Mitochondria were isolated from harvested cells using a mitochondria/cytosol Fractionation Kit (Beyotime Institute of Biotechnology, Shanghai, China). Calcium concentration in mitochondrial cytoplasm was determined with the calcium sensitive dye fluo-3; it was dissolved in dimethylsulfoxide to give a stock solution of 1 mg/mL and stored at -20 °C in the dark.

### Transmission electron microscopy

Cultured bMECs in six-well plates were challenged with HB-AF5 or HLJ-D2 *K. pneumoniae* (MOI = 5), with noninfected bMECs similarly incubated as a control. At 6 hpi, bMECs were washed with PBS (pH 7.2), fixed with 2% glutaraldehyde and 1% paraformaldehyde (pH 7.2; Sinopharm Chemical Reagent Co., Shanghai, China) for 45 min at room temperature and processed for TEM [15]. After washing with PBS, the fixed cells were harvested with a rubber scraper (Thermo Fisher Scientific). Then, cells were dehydrated using graded ethanol and acetone (three changes, 10 min each) and sequentially embedded in epoxy resin acetone mixtures (2:1) for 2 h and then in pure resin overnight at 37 °C. After resin had polymerized, ultra-thin sections were cut (Leica EM, Wetzlar, Germany), stained with 1% uranyl acetate followed by lead citrate, and viewed with a transmission electron microscope (Hitachi H-7650) at 80 kV. Imaging was performed using a 4 k CCD camera (Gatan Inc., Pleasanton, CA, USA) and iTEM software.

### Statistical analyses

For assessment of MDA, T-AOC, ROS, MMP, cytoplasmic and mitochondrial Ca^2+^ concentrations, results were obtained from three independent experiments. Two-way ANOVA was used to determine effects of group and time, and their interaction, and Duncan’s multiple range test used to locate significant differences. A paired Student’s *t*-test with a 95% confidence interval was used to make contemporaneous comparisons between HLJ-D2 versus HB-AF5 groups. All statistical analyses were done with Statistical Product and Service Solutions 20.0 software (SPSS, Inc., Chicago, IL, USA) and *P* < 0.05 was considered significant. Results are expressed as mean ± standard deviation (SD). Data were displayed with Graphpad Prism V6.0 (Data Analysis and Graphing Software, San Diego, CA, USA). Data designated as independent variables in the model were as follows: treatments (time points or by isolates with HLJ-D2 or HB-AF5) in the MDA, T-AOC, ROS measurement assays, MMP assays, and cytoplasmic and mitochondrial Ca^2+^ concentration assays.

## Results

### K. pneumoniae up- or down-regulated DEGs associated with mitochondrial damage in bMECs

#### GO enrichment analysis of DEGs

**Figure 1:**
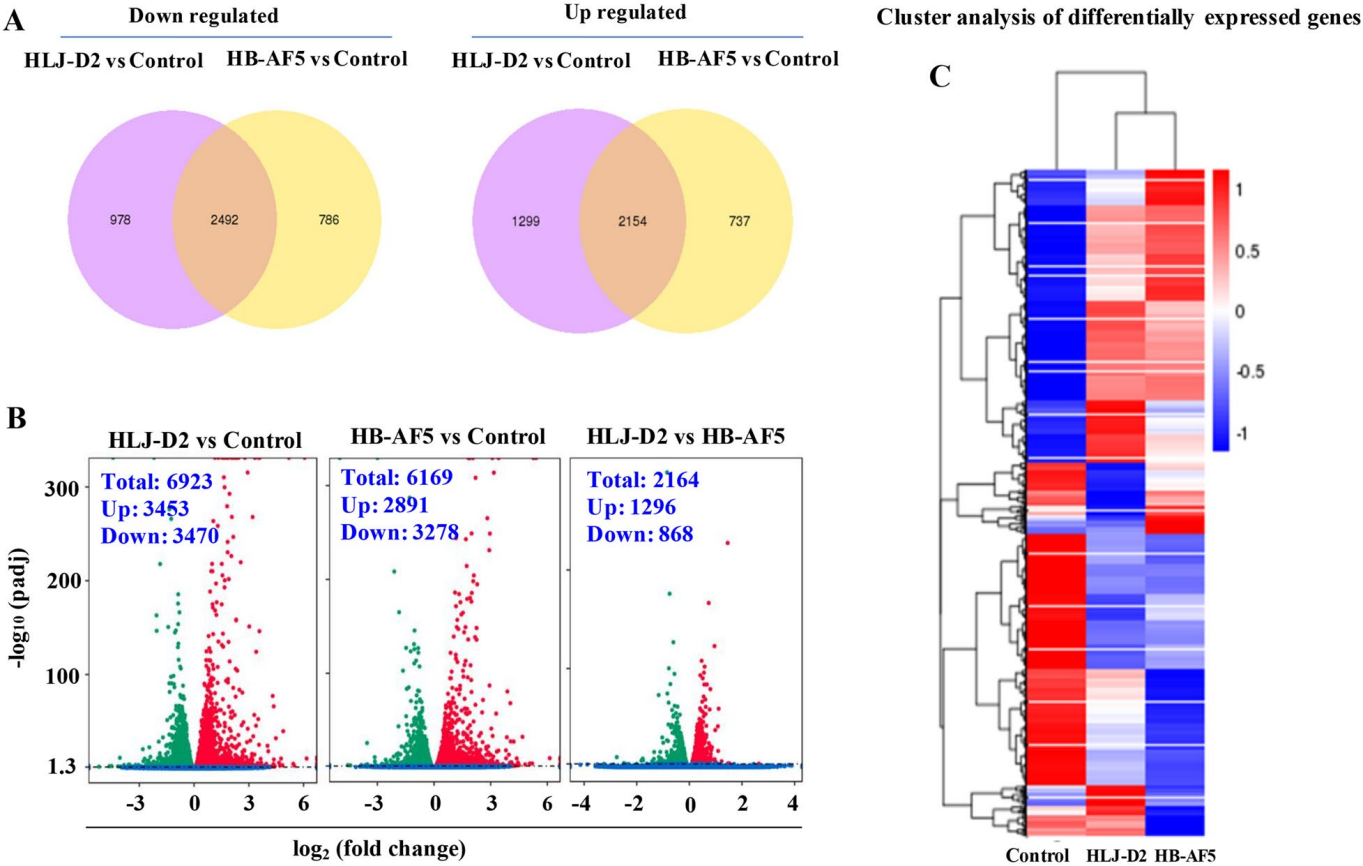
**Transcriptome analysis of differential expression genes (DEGs) in K. pneumoniae-infected bovine mammary epithelial cells (bMECs). A** Venn diagrams showed the total number of up- and down-regulated DEGs in bMECs infected with *K. pneumoniae* (strain HLJ-D2 or HB-AF5) compared to non-infected bMECs at 6 hpi. **B** Volcano plots showed the DEGs in bMECs induced by infection with *Klebsiella pneumoniae* (strain HLJ-D2 or HB-AF5) compared to non-infected bMECs at 6 hpi, as analyzed by the DESeq R package. Each point represents one gene. **C** Cluster analysis of DEGs in bMECs infected with *Klebsiella pneumoniae* (strain HLJ-D2 or HB-AF5) compared to non-infected bMECs at 6 hpi.

A total of 6923 and 6169 genes were differentially expressed with fold-change ≥ 2 or ≤ 0.5 (*P*-value < 0.05) after infection with HLJ-D2 or HB-AF5 *K. pneumoniae* (Figure 1A, B). The HLJ-D2 strain caused more changes at the transcriptional level in bMECs. There were 3453 genes up-regulated and 3470 down-regulated for the HLJ-D2 strain, whereas for the HB-AF5 strain, only 2891 genes were up-regulated and 3278 down-regulated. Based on cluster pattern analysis, DEGs from *K. pneumonia*-treated groups (HLJ-D2 and HB-AF5) had higher differential expression patterns compared to the control (Figure 1C).

### Metabolic pathways

**Figure 2:**
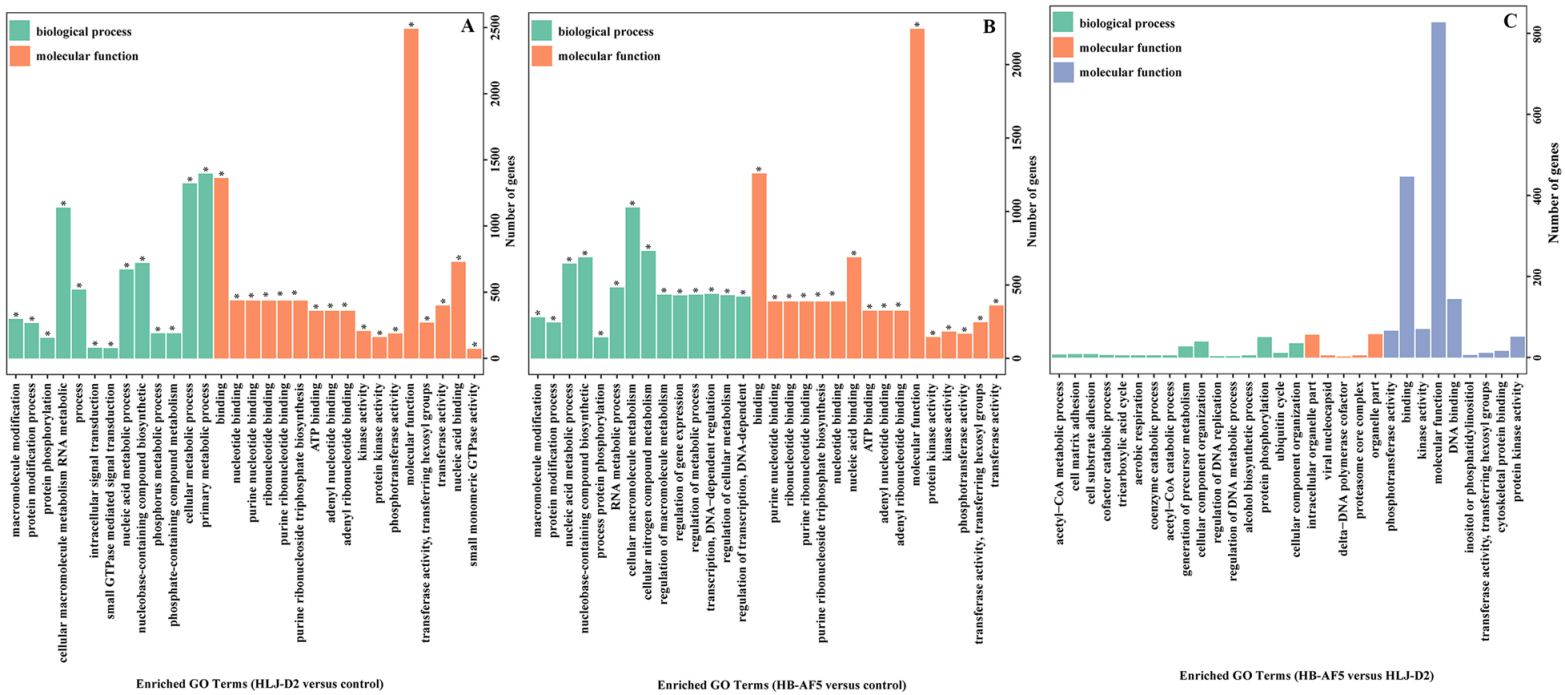
**Enriched GO terms in** ***K. pneumoniae*-infected bovine mammary epithelial cells (bMECs). A** Enriched GO terms in HLJ-D2 *Klebsiella pneumoniae*-infected bMECs versus non-infected bMECs at 6 hpi*.* **B** Enriched GO terms in HB-AF5 *Klebsiella pneumoniae*-infected bMECs versus non-infected bMECs at 6 hpi. **C** Enriched GO terms in HLJ-D2 *Klebsiella pneumoniae*-infected bMECs versus HB-AF5 *Klebsiella pneumoniae*-infected bMECs at 6 hpi.

**Figure 3:**
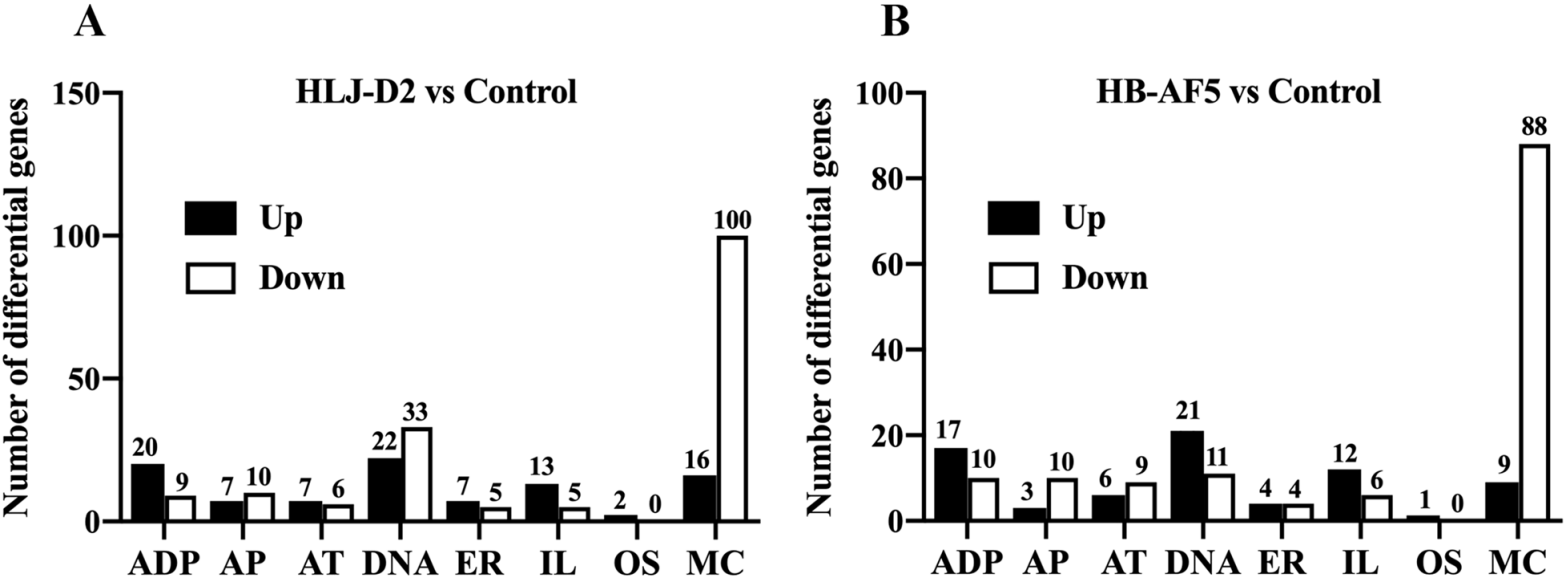
**Differentially expressed genes (DEGs) in** ***K. pneumoniae***-**infected bovine mammary epithelial cells (bMECs). A** Number of DEGs related to ADP, apoptosis (AP), autophagy (AT), DNA, endoplasmic reticulum (ER), interleukin (IL), oxidative stress (OS) and mitochondria (MC) in bMECs infected by HLJ-D2 *K. pneumoniae*. **B** Number of DEGs related to ADP, AP, AT, DNA, ER, IL, OS, and MC in bMECs infected by HB-AF5 *K. pneumoniae.*

In comparison to the control group, the top three enriched functional terms were cellular macromolecule metabolism, binding and molecular function in bMECs incubated with HB-AF5, whereas for HLJ-D2, they were primary metabolic process, binding and molecular function (Figure 2). Compared to the control, the most common up-regulated DEGs in bMECs after HLJ-D2 infection were associated with DNA (22), ADP (20), mitochondria (16) and interleukin (13), whereas mitochondria (100) and DNA (33) were also frequently detected as down-regulated DEGs (Figure 3A). For bMECs infected with the HB-AF5 strain, the most common up-regulated DEGs were DNA (21), ADP (17) and interleukin (12), whereas the most frequently down-regulated DEGs were mitochondria (88) and DNA (11) (Figure 3B).

### K. pneumoniae induced oxidative damage in bMECs

**Figure 4:**
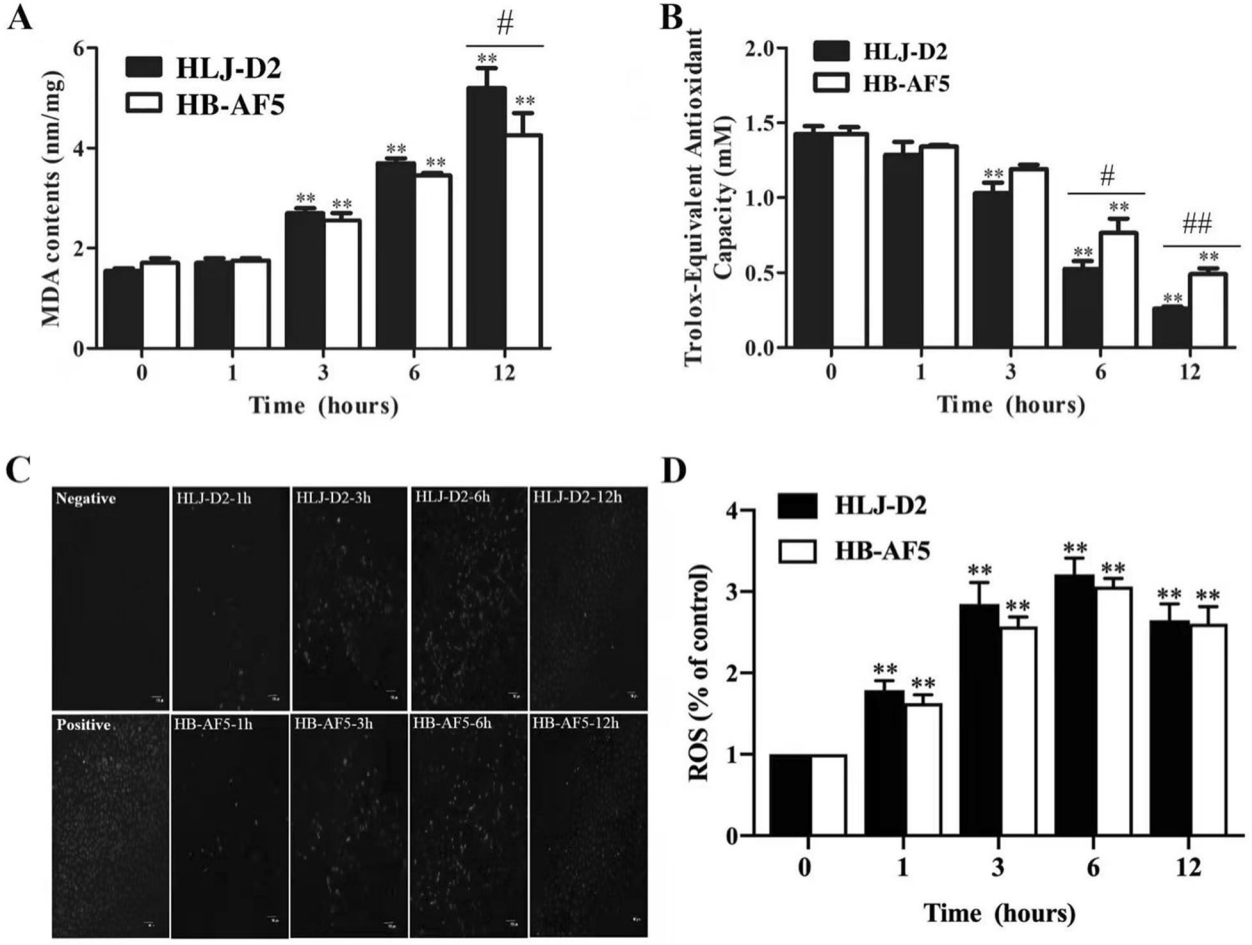
**Effects of** ***Klebsiella pneumoniae*** **on oxidative response in bovine mammary epithelial cells (bMECs)**. bMECs incubated with *Klebsiella pneumoniae* (strain HLJ-D2 and HB-AF5) for 0, 1, 3, 6 and 12 h were collected for assessment of malondialdehyde (MDA) (**A)**, total antioxidant capacity (T-AOC) (**B)** and ROS concentrations (**C)**. **C** Concentrations of ROS were assessed using fluorescence microscopy. **D** Concentrations of ROS were quantified with Image J software. Data are mean ± SD of three independent experiments. Statistical significance indicated by: ∗ *P* < 0.05, ∗  ∗ *P* < 0.01 for comparisons of HLJ-D2 and HB-AF5 to the control group, and ^#^*P* < 0.05, ^##^*P* < 0.01 for comparisons between HLJ-D2 and HB-AF5 at the same time point.

Compared to control, the MDA concentration in bMECs incubated with *K. pneumoniae* was not significantly different at 1 hpi, but was higher at 3, 6 and 12 hpi (*P* < 0.05). However, there was no significant difference between the two strains for MDA concentrations (Figure 4A). Furthermore, compared to non-infected cells at 0 h, T-AOC concentrations in infected groups were not significantly different at 1 hpi, but lower than the control at 3, 6 and 12 hpi (*P* < 0.05), with no significant difference between the two strains (Figure 4B). Production of ROS in bMECs incubated with *K. pneumoniae* increased at 3, 6 and 12 hpi (*P* < 0.05; Figure 4D).

### K. pneumoniae decreased MMP in bMECs

**Figure 5:**
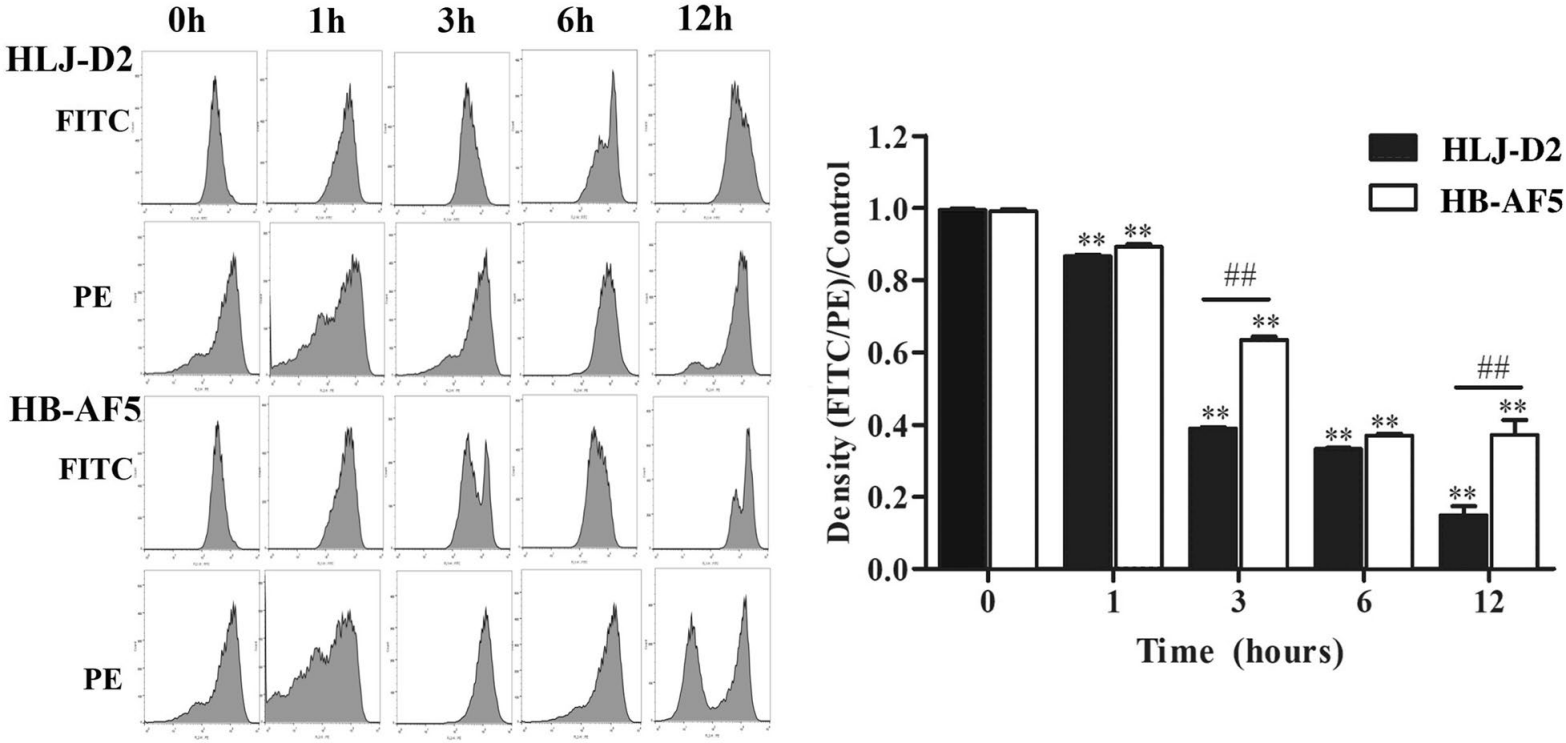
**Effects of** ***Klebsiella pneumoniae*** **on mitochondrial membrane potential (MMP) in bovine mammary epithelial cells (bMECs)**. bMECs incubated with *Klebsiella pneumoniae* (strain HLJ-D2 or HB-AF5) for 0, 1, 3, 6 and 12 h were collected for assessment of mitochondrial membrane potential (MMP) using JC-1 staining. Data are mean ± SD of three independent experiments. FITC: Green light channel, PE: red light channel. Statistical significance indicated by: ∗ *P* < 0.05, ∗  ∗ *P* < 0.01 for comparisons of HLJ-D2 and HB-AF5 to the control group, and ^#^*P* < 0.05, ^##^*P* < 0.01 for comparisons between HLJ-D2 and HB-AF5 at the same timepoint.

The ratio of MMP between the infection groups and control was calculated to determine MMP changes in bMECs. Compared to control, MMP was lower at 1, 3, 6 and 12 hpi (*P* < 0.05) in bMECs incubated with either *K. pneumoniae* strain (Figure 5). In addition, at 3 and 12 hpi, MMP was lower (*P* < 0.05) in bMECs infected with HLJ-D2 versus those infected with HB-AF5.

### K. pneumoniae increased cytoplasmic and mitochondrial calcium concentrations

**Figure 6:**
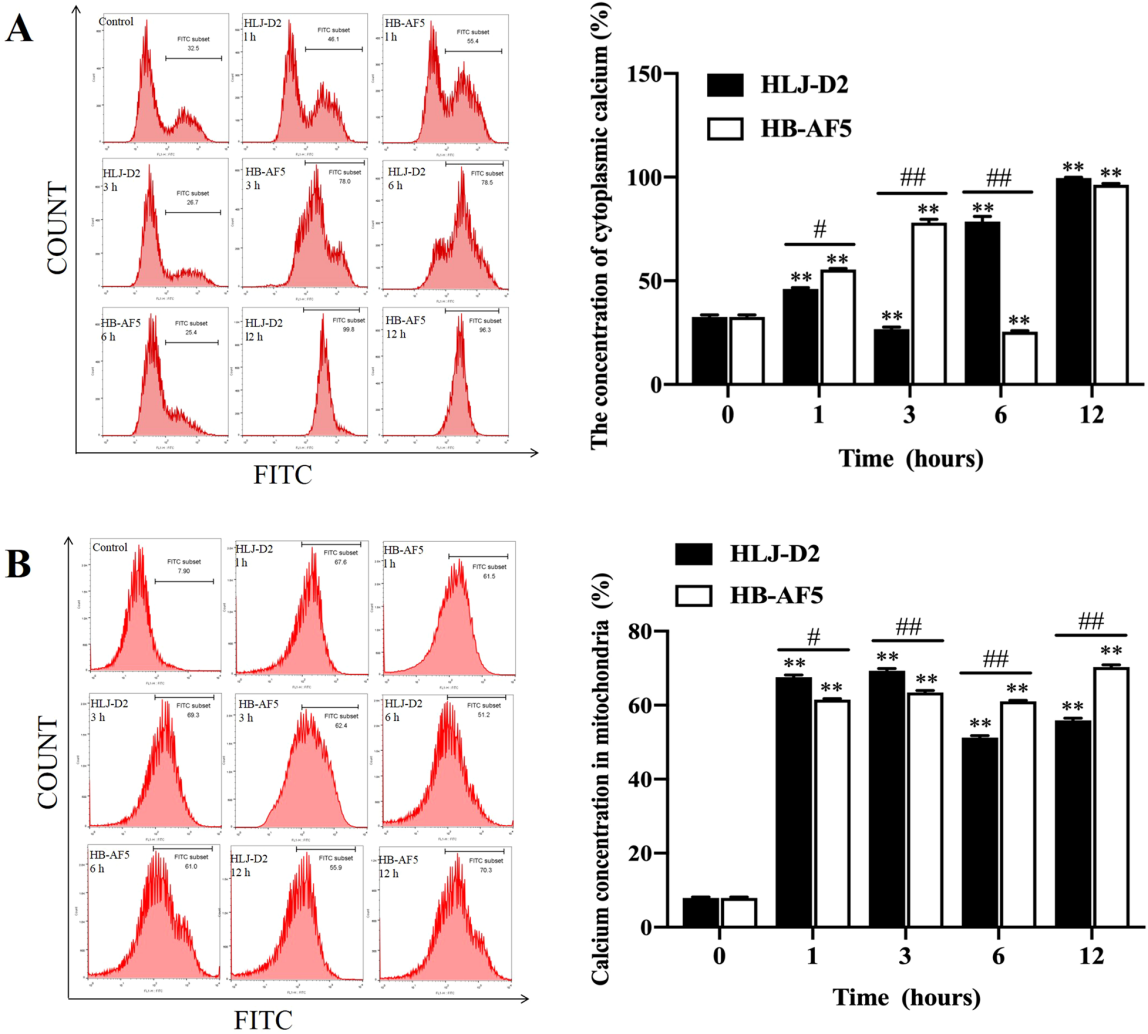
**Effects of** ***Klebsiella pneumoniae*** **on calcium concentrations of bovine mammary epithelial cells (bMECs)**. bMECs incubated with *Klebsiella pneumoniae* (strain HLJ-D2 or HB-AF5) for 0, 1, 3, 6 and 12 h were collected for assessment of cytoplasmic **A** and mitochondrial **B** calcium concentrations using fluo-3 staining. Data are mean ± SD of three independent experiments. Statistical significance indicated by: ∗ *P* < 0.05, ∗  ∗ *P* < 0.01 for the comparison of HLJ-D2 and HB-AF5 to the control group, and ^#^*P* < 0.05, ^##^*P* < 0.01 for the comparison between HLJ-D2 and HB-AF5 at the same timepoint.

Compared to the control, cytoplasmic calcium concentrations in bMECs were generally higher after incubation with strains HLJ-D2 or HB-AF5 (*P* < 0.05), with differences between the two *K. pneumoniae* infection groups (*P* < 0.05), except for 12 hpi (Figure 6A). Meanwhile, there were huge increases in mitochondrial calcium concentrations in bMECs at 1, 3, 6 and 12 hpi, in both infection groups (Figure 6A). In addition, mitochondrial calcium concentrations were higher (*P* < 0.01) in bMECs infected with HLJ-D2 compared to HB-AF5 at 1 and 3 hpi, whereas at 6 and 12 hpi, mitochondrial calcium concentrations were higher (*P* < 0.01) in bMECs infected with HB-AF5 compared to HLJ-D2.

### Mitochondrial morphology of bMECs

**Figure 7:**
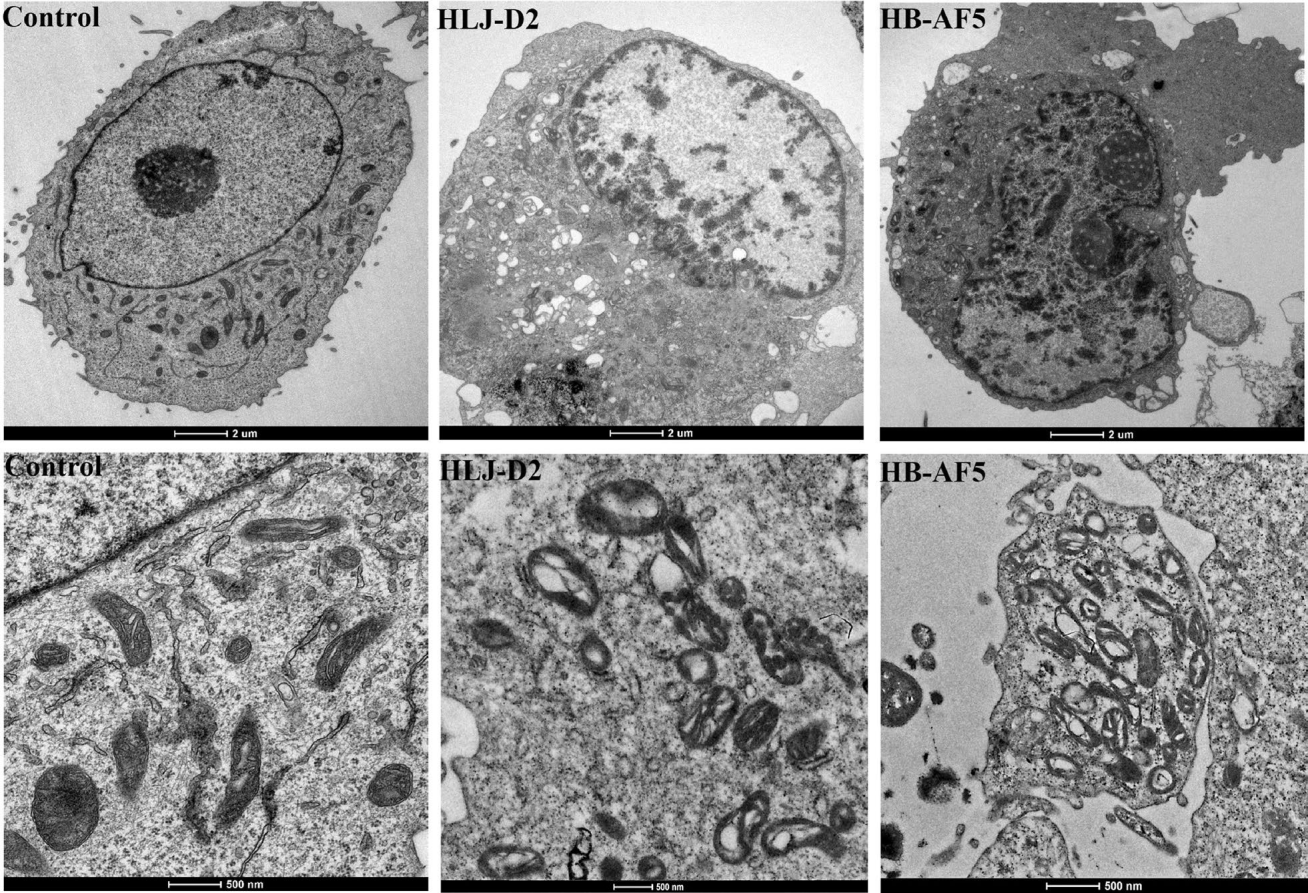
**Effects of** ***Klebsiella pneumoniae*** **on morphological features of mitochondria in bovine mammary epithelial cells (bMECs)**. bMECs incubated with *Klebsiella pneumoniae* (strain HLJ-D2 or HB-AF5) for 6 h were collected for assessment (transmission electron microscopy) of mitochondria.

When viewed with TEM, mitochondria of non-infected bMECs had a normal structure, whereas bMECs infected with *K. pneumoniae* had apparent mitochondrial swelling (Figure 7). At 6 hpi, both strains had caused mitochondria to rupture and cristae to degenerate, followed by severe mitochondrial swelling and vacuolation, with damaged mitochondria becoming increasingly round. The mitochondrial matrix was compressed and dense, and the ridge was shortened, decreased or even disappeared. Additionally, matrix particles had disappeared and the matrix became lighter, whereas the gap in the mitochondrial ridge was wider and cavities of various sizes had formed.

## Discussion

In a previous study, we demonstrated that *K. pneumoniae* was cytopathogenic to bovine mammary epithelial cells, inducing cell damage and apoptosis [15]. Mitochondrial damage has an important role in many kinds of cellular damage, particularly in apoptosis [22, 23]. In the present study, there were alterations in MMP, oxidative stress, and Ca^2+^ concentrations, all known markers of mitochondrial function or damage, consistent with a previous study that determined that cell damage was induced by stimulating mitochondrial dysfunction and calcium dysregulation [24]. Furthermore, substantial morphological damage to mitochondria was confirmed with TEM. Therefore, the present findings provided further evidence that mitochondria may have a key role in damaging bMECs after *K. pneumoniae* infection. We inferred that cellular and molecular changes, including oxidative stress, dysregulation of Ca^2+^ and decreased MMP, were indicators of mitochondrial dysfunction and were involved in damage in bMECs caused by *K. pneumoniae* infection.

Transcriptome analysis, including GO enrichment analysis and pathway enrichment analysis, was done as a systemic screening of up or down-regulated genes in bMECs after infection with two strains of *K. pneumoniae*. Whole transcriptome analysis has been used to explore DEGs that may be involved in particular biological processes in bMECs infected by *K. pneumoniae* [25, 26]. In the present study, DEGs associated with mitochondria were the most common DEGs enriched in bMECs after infection with *K. pneumoniae*, indicating that *K. pneumoniae* had appreciable impacts on mitochondrial functions. Among DEGs associated with ADP, apoptosis, autophagy, DNA, endoplasmic reticulum, interleukin, oxidative stress and mitochondria, changes were greatest in mitochondrial DEGs.

Under various pathological conditions, mitochondria often undergo several morphological changes, including swelling, hyperplasia, hypertrophy and increased density of the mitochondrial matrix [27]. In this study, there was severe mitochondrial swelling and vacuolation, as well as mitochondrial rupture and degenerate cristae after *K. pneumoniae* infection, consistent with a previous study [28] and providing morphological evidence of mitochondrial damage.

Mitochondria are major sources of cellular ROS and key players in oxidative stress, as they are major regulators of cellular energy metabolism [29–31]. When ROS exceed antioxidant capacity of cells, total antioxidative capacity decreases and MDA formation increases, which can accelerate mitochondrial damage [29, 32]. Consistent with a previous study on *Prototheca zopfii* [33], ROS formation increased significantly after *K. pneumoniae* infection, manifested as increased MDA and decreased T-AOC*.* Therefore, we concluded that *K. pneumoniae* provoked ROS production and oxidative stress generation in bMECs. It was suggested that bovine mastitis caused oxidative stress of mammary tissue, manifested as increases in ROS, MDA and decreased T-AOC, resulting in mitochondria-mediated apoptosis and inflammation [33, 34].

The significant decrease in MMP at 1, 3, 6 and 12 hpi indicated that *K. pneumoniae* rapidly and profoundly suppressed MMP. It is noteworthy that MMP is reflective of the functional metabolic status of mitochondria, and a normal MMP is a prerequisite for maintaining mitochondrial metabolism [35]. Therefore, it arguably is a reliable indicator of mitochondrial function. A change in mitochondrial membrane potential can reflect the permeability of mitochondrial inner membrane, an indicator of mitochondrial function and damage. Furthermore, a decreased mitochondrial inner membrane potential can induce apoptosis [27]. Perhaps the reduction in MMP was due to a primary effect of an unknown *Klebsiella* virulence factor.

It is well established that Ca^2+^ has a fundamental role in several cellular functions, regulating or inducing many processes, including proliferation, differentiation, secretion, metabolism, cell death and survival, migration, and gene expression [36]. In this study, infection with *K*. *pneumoniae* increased (*P* < 0.05) Ca^2+^ concentrations in mitochondria of bMECs compared to Ca^2+^ concentrations in mitochondria of non-infected bMECs. Therefore, we concluded that *K*. *pneumoniae* stimulated excessive calcium transfer in bMECs. Excessive cytoplasmic calcium concentrations caused calcium overload in mitochondria, as mitochondria are cytosolic Ca^2+^ buffers [37]. Our results were consistent with a previous study that disruption of Ca^2+^ homeostasis or mitochondrial signaling leads to cell damage [38]. For cytoplasmic Ca^2+^, there was a significant decrease at 3 hpi with HLJ-D2, whereas a significant decrease occurred at 6 hpi with HB-AF5. Subsequently, cytoplasmic Ca^2+^ concentrations increased significantly for both HLJ-D2 and HB-AF5 infected bMECs. Dysregulated Ca^2+^ release from intracellular reservoirs, combined with an influx from the extracellular domain, cause a sustained increase in intracellular Ca^2+^ over time [24]. Perhaps *K. pneumoniae* infection increased membrane permeability in bMECs, leading to a decrease in the Ca^2+^ exchange in the cytoplasm and therefore, a temporary decrease of Ca^2+^ in the cytoplasm. Cytoplasmic Ca^2+^ concentrations are a dynamic system in determining both cell survival and death [37]. A further investigation of the exact mechanisms explaining how *K. pneumoniae* contributes to the dysregulation of Ca^2+^ homeostasis is warranted.

The two *K. pneumoniae* strains (non-HMV and HMV) were also used in our previous study [15]. Compared to the non-HMV strain (HB-AF5), the HMV strain (HLJ-D2) had more up- or down-regulated DEGs, and a significantly greater effect on mitochondrial calcium concentrations. However, the non-HMV strain caused a bigger change in MMP. Therefore, we assumed that there were differences among *K. pneumoniae* strains in their ability to damage mitochondria. However, the HMV phenotype of *K. pneumoniae* did not have a key role in causing mitochondrial damage. We emphasize that the results of our in vitro study cannot be generalized to represent in vivo pathology induced by *K. pneumoniae* in the bovine udder.

There were many DEGs associated with mitochondria in bMECs after infection with *K. pneumoniae*. Furthermore, *K. pneumoniae* infection significantly decreased MMP and increased ROS generation, whereas cytoplasmic and mitochondrial Ca^2+^ in bMECs were significantly increased. Therefore, based on multiple end points, *K. pneumoniae* induced profound mitochondrial damage and dysfunction in bMECs. Furthermore, we inferred that this contributed to the pathogenicity of cellular damage in CM in dairy cows induced by *K. pneumoniae*.

## Data Availability

All data generated or analyzed during this study are included in this published article.

## Abbreviations

ABTS: 2,2′-Azino-bis (3-ethylbenzthiazoline-6-sulfonic acid)
bMECs: Bovine mammary epithelial cells;
CM: Clinical mastitis
DCFH-DA: Dichlorodihydrofluorescein diacetate
DEGs: Differentially expressed genes
FBS: Fetal bovine serum
GO: Gene Ontology
HMV: Hypermucoviscous
hpi: Hours post-infection
IL-1β: Interleukin-1β
IL-6: Interleukin-6
IL-8: Interleukin-8
MDA: Malondialdehyde
MOI: Multiplicity of infection
MMP: Mitochondrial membrane potential
PBS: Phosphate Buffered Saline
ROS: Reactive oxygen species
SD: Standard deviation
SPSS: Statistical Product and Service Solutions
T-AOC: Total antioxidant capacity
TBA: Thiobarbituric acid
TEM: Transmission electron microscopy
TNF-α: Tumor necrosis factor-α

## References

[1] Heikkilä AM, Liski E, Pyörälä S, Taponen S (2018). Pathogen-specific production losses in bovine mastitis. J Dairy Sci.

[2] Gao J, Barkema HW, Zhang L, Liu G, Deng Z, Cai L, Shan R, Zhang S, Zou J, Kastelic JP, Han B (2017). Incidence of clinical mastitis and distribution of pathogens on large Chinese dairy farms. J Dairy Sci.

[3] Schukken Y, Chuff M, Moroni P, Gurjar A, Santisteban C, Welcome F, Zadoks R (2012). The “other” gram-negative bacteria in mastitis: *Klebsiella*, *Serratia*, and more. Vet Clin North Am Food Anim Pract.

[4] Fuenzalida MJ, Ruegg PL (2019). Negatively-controlled, randomized clinical trial to evaluate intramammary treatment of nonsevere, gram-negative clinical mastitis. J Dairy Sci.

[5] Rainard P, Cunha P, Bougarn S, Fromageau A, Rossignol C, Gilbert FB, Berthon P (2013). T helper 17-associated cytokines are produced during antigen-specific inflammation in the mammary gland. PLoS One.

[6] Thompson-Crispi K, Atalla H, Miglior F, Mallard BA (2014). Bovine mastitis: frontiers in immunogenetics. Front Immunol.

[7] West AP, Shadel GS, Ghosh S (2011). Mitochondria in innate immune responses. Nat Rev Immunol.

[8] Giorgi C, Baldassari F, Bononi A, Bonora M, De Marchi E, Marchi S, Missiroli S, Patergnani S, Rimessi A, Suski JM, Wieckowski MR, Pinton P (2012). Mitochondrial Ca (2+) and apoptosis. Cell Calcium.

[9] Krysko DV, Agostinis P, Krysko O, Garg AD, Bachert C, Lambrecht N, Vandenabeele P (2011). Emerging role of damage-associated molecular patterns derived from mitochondria in inflammation. Trends Immunol.

[10] Rongvaux A (2017). Innate immunity and tolerance toward mitochondria. Mitochondrion.

[11] Spier A, Stavru F, Cossart P (2019) Interaction between intracellular bacterial pathogens and host cell mitochondria. Microbiol Spectr 7:BAI-0016–2019 10.1128/microbiolspec.bai-0016-2019 PMC11590420 30848238

[12] Gómez-Crisóstomo NP, López-Marure R, Zapata E, Zazueta C, Martínez-Abundis E (2013). Bax induces cytochrome C release by multiple mechanisms in mitochondria from MCF7 Cells. J Bioenerg Biomembr.

[13] Wang Z, Wang J, Xie R, Liu R, Lu Y (2015). Mitochondria-derived reactive oxygen species play an important role in doxorubicin-induced platelet apoptosis. Int J Mol Sci.

[14] Suski JM, Lebiedzinska M, Bonora M, Pinton P, Duszynski J, Wieckowski MR (2012). Relation between mitochondrial membrane potential and ROS formation. Methods Mol Biol.

[15] Cheng J, Zhang J, Han B, Barkema HW, Cobo ER, Kastelic P, Zhou M, Shi Y, Wang J, Yang R, Gao J (2020). *Klebsiella pneumoniae* isolated from bovine mastitis is cytopathogenic for bovine mammary epithelial cells. J Dairy Sci.

[16] Gao J, Li S, Zhang J, Zhou Y, Xu S, Barkema HW, Nobrega DB, Zhu C, Han B (2019). Prevalence of potential virulence genes in *Klebsiella* spp. isolated from cows with clinical mastitis on large Chinese dairy farms. Foodborne Pathog Dis.

[17] Ferreira JA (2007). The Benjamini-Hochberg method in the case of discrete test statistics. Int J Biostat.

[18] Young MD, Wakefield MJ, Smyth GK, Oshlack A (2010). Gene ontology analysis for RNA-seq: accounting for selection bias. Genome Biol.

[19] Thimm O, Bläsing O, Gibon Y, Nagel A, Meyer S, Krüger P, Selbig J, Müller LA, Rhee SY, Stitt M (2004). MAPMAN: A user-driven tool to display genomics data sets onto diagrams of metabolic pathways and other biological processes. Plant J.

[20] Warnes GR, Bolker B, Bonebakker L, Gentleman R, Huber W, Liaw A, Lumley T, Maechler M, Magnusson A, Moeller S, Schwartz M, Venables B, Galili T (2020) gplots: Various R programming tools for plotting data. R package version 3.0.4. https://CRAN.R-project.org/package=gplots

[21] Thomas PD (2017). The Gene Ontology and the meaning of biological function. Methods Mol Biol.

[22] Hussain S (2019). Measurement of nanoparticle-induced mitochondrial membrane potential alterations. Methods Mol Biol.

[23] Faas MM, Vos PD (2020). Mitochondrial function in immune cells in health and disease. Biochim Biophys Acta Mol Basis Dis.

[24] Leong HS, Philp M, Simone M, Witting PK, Fu S (2020). Synthetic cathinones induce cell death in dopaminergic SH-SY5Y cells via stimulating mitochondrial dysfunction. Int J Mol Sci.

[25] Domhan S, Schwager C, Wei Q, Muschal S, Sommerer C, Morath C, Wick W, Maercker C, Debus J, Zeier M, Huber PE, Abdollahi A (2014). Deciphering the systems biology of mTOR inhibition by integrative transcriptome analysis. Curr Pharm Des.

[26] Xiu L, Fu YB, Deng Y, Shi XJ, Bian ZY, Ruhan A, Wang X (2015). Deep sequencing-based analysis of gene expression in bovine mammary epithelial cells after *Staphylococcus aureus*, *Escherichia coli*, and *Klebsiella pneumoniae* Infection. Genet Mol Res.

[27] Shahid M, Cobo ER, Chen L, Cavalcante PA, Barkema HW, Gao J, Xu S, Liu Y, Knight CG, Kastelic JP, Han B (2020). *Prototheca zopfii* genotype II induces mitochondrial apoptosis in models of bovine mastitis. Sci Rep.

[28] Chen W, Liu Y, Zhang L, Gu X, Liu G, Shahid M, Gao J, Ali T, Han B (2017). *Nocardia cyriacigeogica* from bovine mastitis induced in vitro apoptosis of bovine mammary epithelial cells via activation of mitochondrial-caspase pathway. Front Cell Infect Microbiol.

[29] Dan Dunn J, Alvarez LA, Zhang X, Soldati T (2015). Reactive oxygen species and mitochondria: a nexus of cellular homeostasis. Redox Biol.

[30] Barbosa DJ, Capela JP, Feio-Azevedo R, Teixeira-Gomes A, Bastos MDL, Carvalho F (2015). Mitochondria: Key players in the neurotoxic effects of amphetamines. Arch Toxicol.

[31] Adam-Vizi V, Chinopoulos C (2006). Bioenergetics and the formation of mitochondrial reactive oxygen species. Trends Pharmacol Sci.

[32] Metcalfe NB, Alonso-Alvarez C (2010). Oxidative stress as a life-history constraint: the role of reactive oxygen species in shaping phenotypes from conception to death. Funct Ecol.

[33] Shahid M, Gao J, Zhou Y, Liu G, Ali T, Deng Y, Sabir N, Su J, Han B (2017). *Prototheca zopfii* isolated from bovine mastitis induced oxidative stress and apoptosis in bovine mammary epithelial cells. Oncotarget.

[34] Fu SC, Liu JM, Lee KI, Tang FC, Fang KM, Yang CY, Su CC, Chen HH, Hsu RJ, Chen YW (2020). Cr (VI) induces ROS-mediated mitochondrial-dependent apoptosis in neuronal cells via the activation of Akt/ERK/AMPK signaling pathway. Toxicol In Vitro.

[35] Teodoro JS, Palmeira CM, Rolo AP (2018). Mitochondrial membrane potential (ΔΨ) fluctuations associated with the metabolic states of mitochondria. Methods Mol Biol.

[36] Giorgi C, Danese A, Missiroli S, Patergnani S, Pinton P (2018). Calcium dynamics as a machine for decoding signals. Trends Cell Biol.

[37] Rizzuto R, De Stefani D, Raffaello A, Mammucari C (2012). Mitochondria as sensors and regulators of calcium signalling. Nat Rev Mol Cell Biol.

[38] Chen X, Xing J, Jiang L, Qian W, Wang Y, Sun H, Wang Y, Xiao H, Wang J, Zhang J (2016). Involvement of calcium/calmodulin-dependent protein kinase II in methamphetamine-induced neural damage: methamphetamine induces neural damage through Ca^2+^ signaling. J Appl Toxicol.

